# The lower airways microbiota and antimicrobial peptides indicate dysbiosis in sarcoidosis

**DOI:** 10.1186/s40168-022-01362-4

**Published:** 2022-10-19

**Authors:** Kristel S. Knudsen, Sverre Lehmann, Rune Nielsen, Solveig Tangedal, Andreu Paytuvi-Gallart, Walter Sanseverino, Einar M. H. Martinsen, Pieter S. Hiemstra, Tomas M. Eagan

**Affiliations:** 1 Department of Clinical Science, University of Bergen, Bergen, Norway; 2 Department of Thoracic Medicine, Haukeland University Hospital, Bergen, Norway; 3 Sequentia Biotech SL, Barcelona, Spain; 4 Department of Pulmonology, Leiden University Medical Center, Leiden, Netherlands

## Abstract

**Background:**

The role of the pulmonary microbiome in sarcoidosis is unknown. The objectives of this study were the following: (1) examine whether the pulmonary fungal and bacterial microbiota differed in patients with sarcoidosis compared with controls; (2) examine whether there was an association between the microbiota and levels of the antimicrobial peptides (AMPs) in protected bronchoalveolar lavage (PBAL).

**Methods:**

Thirty-five sarcoidosis patients and 35 healthy controls underwent bronchoscopy and were sampled with oral wash (OW), protected BAL (PBAL), and left protected sterile brushes (LPSB). The fungal ITS1 region and the V3V4 region of the bacterial 16S rRNA gene were sequenced. Bioinformatic analyses were performed with QIIME 2. The AMPs secretory leucocyte protease inhibitor (SLPI) and human beta defensins 1 and 2 (hBD-1 and hBD-2), were measured in PBAL by enzyme-linked immunosorbent assay (ELISA).

**Results:**

*Aspergillus* dominated the PBAL samples in sarcoidosis. Differences in bacterial taxonomy were minor. There was no significant difference in fungal alpha diversity between sarcoidosis and controls, but the bacterial alpha diversity in sarcoidosis was significantly lower in OW (*p* = 0.047) and PBAL (*p* = 0.03) compared with controls. The beta diversity for sarcoidosis compared with controls differed for both fungi and bacteria. AMP levels were significantly lower in sarcoidosis compared to controls (SLPI and hBD-1: *p* < 0.01). No significant correlations were found between alpha diversity and AMPs.

**Conclusions:**

The pulmonary fungal and bacterial microbiota in sarcoidosis differed from in controls. Lower antimicrobial peptides levels were seen in sarcoidosis, indicating an interaction between the microbiota and the innate immune system. Whether this dysbiosis represents a pathogenic mechanism in sarcoidosis needs to be confirmed in experimental studies.

Video Abstract

**Supplementary Information:**

The online version contains supplementary material available at 10.1186/s40168-022-01362-4.

## Introduction

Sarcoidosis is a systemic inflammatory disease that can be challenging to diagnose and treat. Disease manifestations are heterogeneous, with symptoms varying considerably [1]. The disease resolves or becomes chronic, seemingly independent of treatment. Virtually all organs may be affected, but the lungs by far most commonly.

The causative agents inducing the immune response in sarcoidosis are yet somewhat of an enigma, but the immune system seems to respond to different substances by producing inflammatory cells organized in granulomas. It has long been recognized that the immune response could reflect an infectious etiology and scientists have for decades searched for that one pathogen that might cause sarcoidosis [1, 2]. Despite previous efforts, no single candidate has been discovered as the main microbial trigger for sarcoidosis; however, the impact of nontuberculous *Mycobacterium* and *Propionibacterium* has been widely studied [2, 3].

Novel culture independent techniques as high-throughput 16S rRNA gene sequencing has revealed the presence of a diverse bacterial microbiota in the lungs [4]. In sarcoidosis, only two studies to our knowledge have been published on the pulmonary fungal microbiota and they both detected enrichment of *Aspergillus* [5, 6]. A small number of studies have investigated the pulmonary bacterial microbiota in sarcoidosis, exploring whether the sarcoid lower airways microbiome was different from healthy controls [5, 7, 8] or other patient groups [9–12]. The results have been inconsistent, with three studies finding differences [8, 11, 12] and four not [5, 7, 9, 10].

Antimicrobial peptides and proteins (AMPs) are key effector molecules in the innate immune system against respiratory pathogens [13, 14]. There is only one scientific report that describes a possible connection between levels of AMPs in bronchoalveolar lavage and sarcoidosis [15], and no prior studies of the microbiota and AMPs in sarcoidosis have been conducted.

The aim of this bronchoscopy study was to examine whether the fungal and bacterial pulmonary microbiota differed in patients with sarcoidosis compared to healthy controls, and if there was an association between the microbiota and the AMPs.

## Methods

### Study design

Participants were recruited from our outpatient clinic at the Department of Thoracic Medicine, Haukeland University Hospital, Norway, participating in The Microbiome in Interstitial Lung Disease study (MicroILD) [16] and The Bergen Chronic Obstructive Pulmonary Disease Microbiome study (MicroCOPD) [17]. All study participants were examined in one center with consistent methodology by the same investigators. The MicroILD study included 70 patients in 2014–2016, of which 35 previously diagnosed with sarcoidosis. The sarcoidosis patients were in stable condition (2 patients received a maximum 5 mg/day of prednisolone) and no participants received antibiotics 14 days before bronchoscopy. The MicroCOPD study enrolled 130 COPD patients, 16 asthma patients and 103 healthy controls in 2012–2015. From this study, 35 controls were randomly selected by use of the *runiform ()* function in Stata 14.2 [18]. Metadata included lung function measurements, blood samples, and a standardized interview with medical history, medications, and comorbidities. The Norwegian regional ethical committee approved the MicroILD and MicroCOPD studies with case numbers 2014/1393 and 2011/1307, respectively. Written consent was obtained from all participants.

### Bronchoscopy

The participants underwent bronchoscopy with oral access and topical anesthesia in a supine position. Intravenous alfentanil (0.25–1.0 mg) was offered. To minimize contamination, no suctioning was performed above the vocal cords. During bronchoscopy we collected 3 protected sterile brushes from the left upper lobe (LPSB) and 3 × 50 ml (2 × 50 ml for controls) of bronchoalveolar lavage from the right middle lobe through a sterile protected sheath (PBAL). Sterile phosphate-buffered saline (PBS) was used for all fluid samples, including 10 ml of oral wash (OW). A negative control sample was obtained from the PBS bottle used for the procedural samples of each participant, without being exposed to the participant or the bronchoscope.

### DNA extraction, sequencing, and bioinformatics

The 16S rRNA sequencing was performed during the course of the MicroCOPD and MicroILD studies and included sequencing of more than 2500 samples from almost 400 bronchoscopies, with up to 8 sampling sites per participant. For the ITS sequencing which was performed afterwards, we chose to limit the sequencing to PBAL, OW, and negative control samples, due to cost restraints.

The detailed protocol DNA extraction, PCR and sample preparation for MiSeq sequencing for the 16S rRNA gene analyses is previously published [19]. The Additional file 1 contains a summary together with a detailed account of the sequencing of the fungal ITS1 gene.

The bioinformatic analyses were performed with QIIME 2 [20].

### Cell count and AMPs in PBAL

Cytospin slides were made from PBAL and stained with May-Grünwald-Giemsa. A minimum of 300 cells were counted for differential cell counts by an observer blinded for the patient’s data. The AMPs, SLPI [21], and hBD-1 (PeproTech, London; UK) and hBD-2 (Antigenix America, Melville, NY, USA), were measured in PBAL by enzyme-linked immunosorbent assay (ELISA), performed at the Leiden University Medical Center, the Netherlands.

### Statistical analyses

For statistical analyses we used Stata [18] and QIIME 2 [20]. Continuous variables were analysed as means or medians depending on distribution, and categorical variables as proportions. To investigate differential abundance, we used analysis of composition of microbes (ANCOM) [22]. Faith’s phylogenetic and Shannon’s non-phylogenetic alpha diversity indexes were analysed using Kruskal-Wallis test. Permutational multivariate analysis of variance (PERMANOVA) was used for beta diversities (phylogenetic weighted and unweighted UniFrac, and non-phylogenetic Bray-Curtis and Jaccard). The levels of the AMPs were not normally distributed, and for the hBD-2 several samples had levels below detection limit. Statistical differences in AMPs were calculated with Kruskal-Wallis test, and correlations with alpha diversity tested with Spearman’s correlation tests.

## Results

The demographics of the participants are shown in Table 1. The 35 patients were younger, and less exposed to smoking compared to the 35 healthy controls. The lung function was reduced in sarcoidosis patients as expected. The patients had significantly higher lymphocyte counts in PBAL compared to controls.

**Table 1:** Characteristics of the study population

	Sarcoidosis	Controls	
	*n* = 35	*n* = 35	p*
Male	74 %	49 %	0.03
Age, mean years (SD§)	55.3 (10.9)	66.3 (7.6)	< 0.01
**Pulmonary function, mean % of predicted (SD§)**			
FEV1	85 (15.4)	103 (11.5)	< 0.01
FVC	98 (13.8)	111 (12.5)	< 0.01
DLCO	88 (11.9)	94 (13.1)	0.09
**Smoking habits**			< 0.01
Current smoker	6%	23 %	
Ex smoker	46%	66 %	
Never smoker	48%	11 %	
**BAL cell content %, mean (SD§)**			
Macrophages	71.8 (19.5)	83.6 (11.5)	< 0.01
Neutrophils	4.5 (13.6)	3.8 (4.6)	0.80
Lymphocytes	22.2 (18.7)	12.1 (9.2)	< 0.01
Eosinophils	1.5 (2.3)	0.5 (0.8)	0.02
**Antimicrobial peptides in BAL, median (IQR§)**			
SLPI†, ng/ml	95 (48–175)	187 (144–241)	< 0.01
hBD-1‡, pg/ml	153 (108–331)	329 (235–500)	< 0.01
hBD-2‡, pg/ml	10 (10–10)	10 (10–10)	0.38
**Inflammatory marker in plasma, mean (SD§)**			
Leukocyte particle count (LPK), 10^9/L	5.5 (2.1)	6.5 (2.0)	0.05

*p for differences in sex and smoking habits tested by Pearson chi square. Differences in age, lung function, BAL cell content, and inflammatory markers tested by ANOVA. Differences in antimicrobial peptides tested by Kruskal-Wallis test

^†^Secretory leucocyte protease inhibitor (SLPI)

^‡^human beta defensins 1 and 2 (hBD-1 and hBD-2)

^§^Standard deviation (SD), interquartile range (IQR)

### Taxonomy

**Fig. 1:**
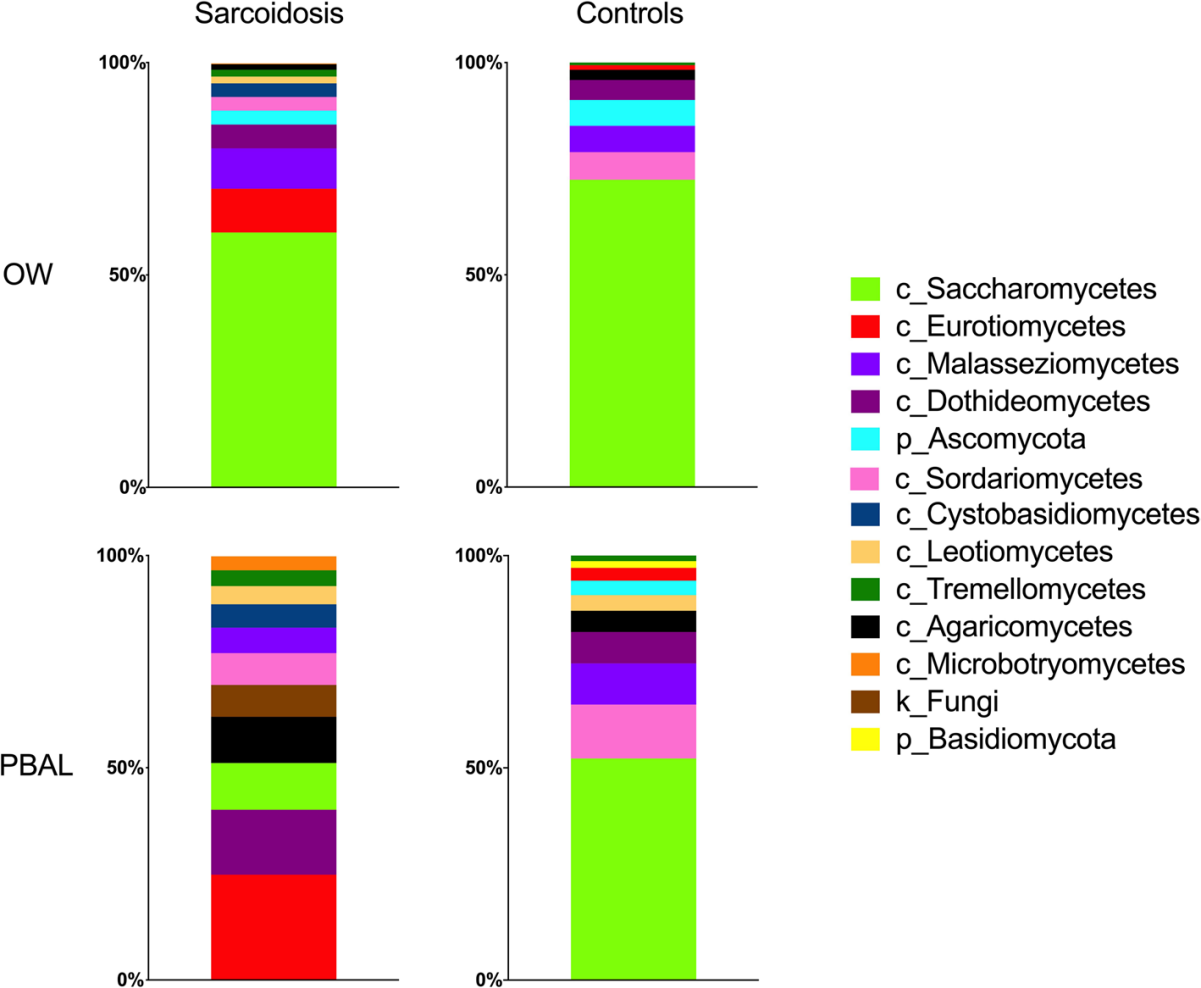
Fungal taxonomy at class level by study groups and sample types (OW: oral wash, PBAL: protected bronchoalveolar lavage)

**Fig. 2:**
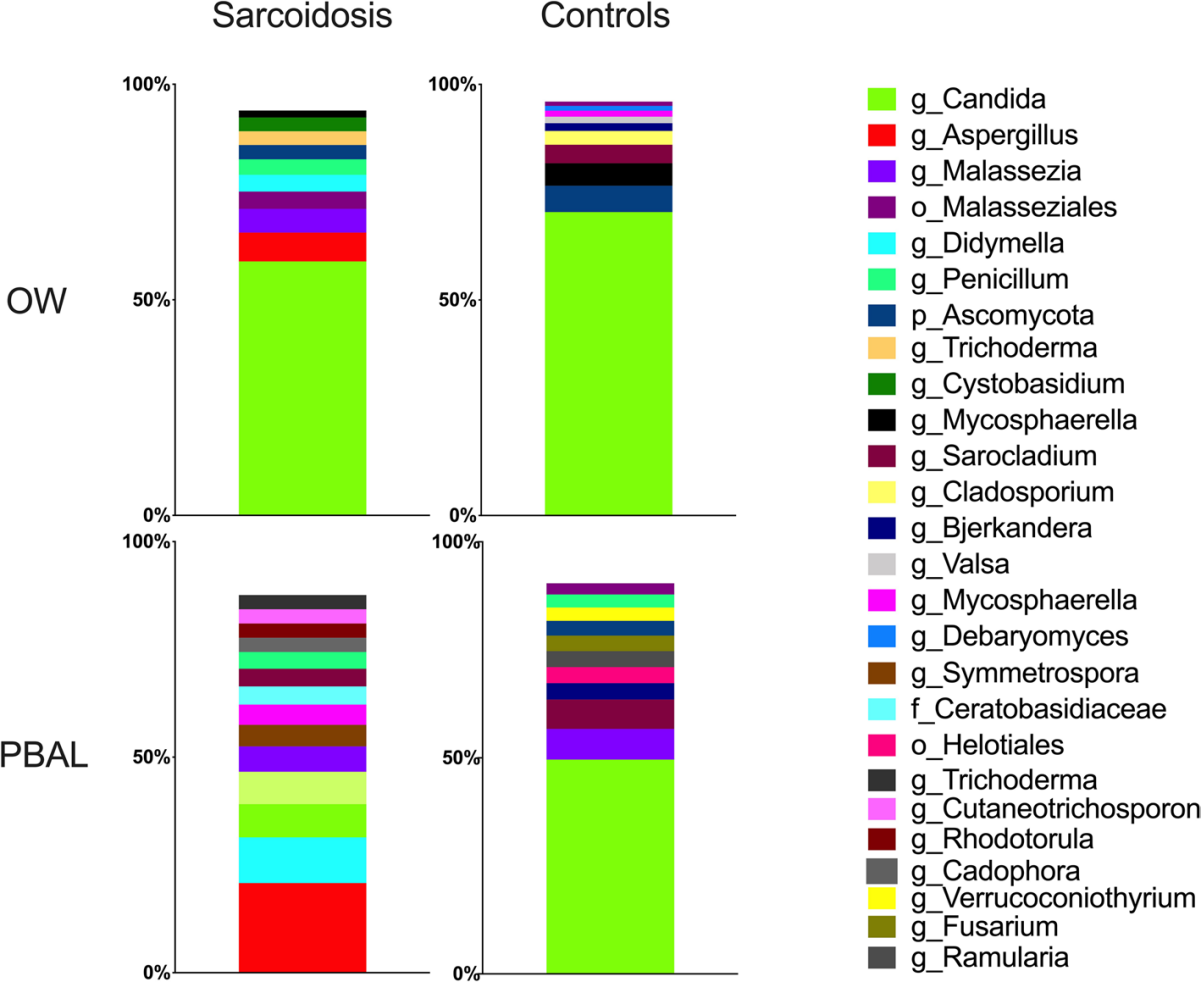
Fungal taxonomy at genus level by study groups and sample types (OW: oral wash, PBAL: protected bronchoalveolar lavage)

The distribution of the fungal taxonomic composition for the different study groups and sample types at class and genus levels respectively are shown with stacked bar plots in Figs. 1 and 2. As the fungal microbiota only contained two phyla, the class level was visualized in addition to genera. Each color represents one taxon and is visualized in the order of decreasing relative abundance in each category. The visual judgement indicated striking differences between the study groups and sample types for the fungi. At class level, *Saccharomycetes* was the most abundant fungal taxa in the OW samples for both study groups and PBAL samples for controls. However, in PBAL samples for sarcoidosis patients, the taxa were more evenly distributed, with the most frequent classes being *Eurotiomycetes* and *Malasseziomycetes.* At genus level, *Candida* was most abundant in the OW samples for both categories, while being less abundant in PBAL samples for patients compared to controls. *Aspergillus* was the most frequent genus measured in PBAL samples for sarcoidosis patients but was undetectable in PBAL and OW samples for controls. ANCOM analysis at class level showed significantly less *Saccharomycetes* in PBAL samples and less *Sordariomycetes* in OW samples from sarcoidosis patients compared to controls (Table 2). ANCOM analysis at genus level verified that *Candida* was significantly less abundant in PBAL samples in patients compared to controls (Table 2).

**Table 2:** An overview of differential abundant taxa by use of ANCOM*

Sarcoidosis vs controls	Fungi		Bacteria		
	OW	PBAL	OW	PBAL	LPSB
Phylum	Ascomycota W1 Fungi W1	ns	Abscondibacteria W1 Fusobacteria W1	Abscondibacteria W1	ns
Class	Sordariomycotes W10	Sacchaomycetes W13	ns	ns	Chitinophagia W4 Bacteroidetes_(C-1) W1 Mollicutes W1 Synergistia W1
Order			ns	ns	Chitinophagales W8
Family			ns	ns	Chitinophagaceae W11
Genus	ns	Candida W31	Stomatobacteria W21	ns	Synergistia W1

*Analysis of Composition of Microbes (ANCOM). Only taxa found to be significantly different (*p* < 0.05) between sarcoidosis patients and controls are listed. ANCOM tests whether pairwise ratios of taxa are different between study groups. The *W* number specifies the number of significantly different ratios. The maximum *W* number for each test is the number of taxa within the group 1. For example, for left protected sterile brush 20 different classes of bacteria was found, and the maximum *W* number would be 19. For Chitinophagia, the ratio between Chitinophagia and 4 other classes were found by ANCOM to be significantly different between sarcoidosis patients and controls

**Fig. 3:**
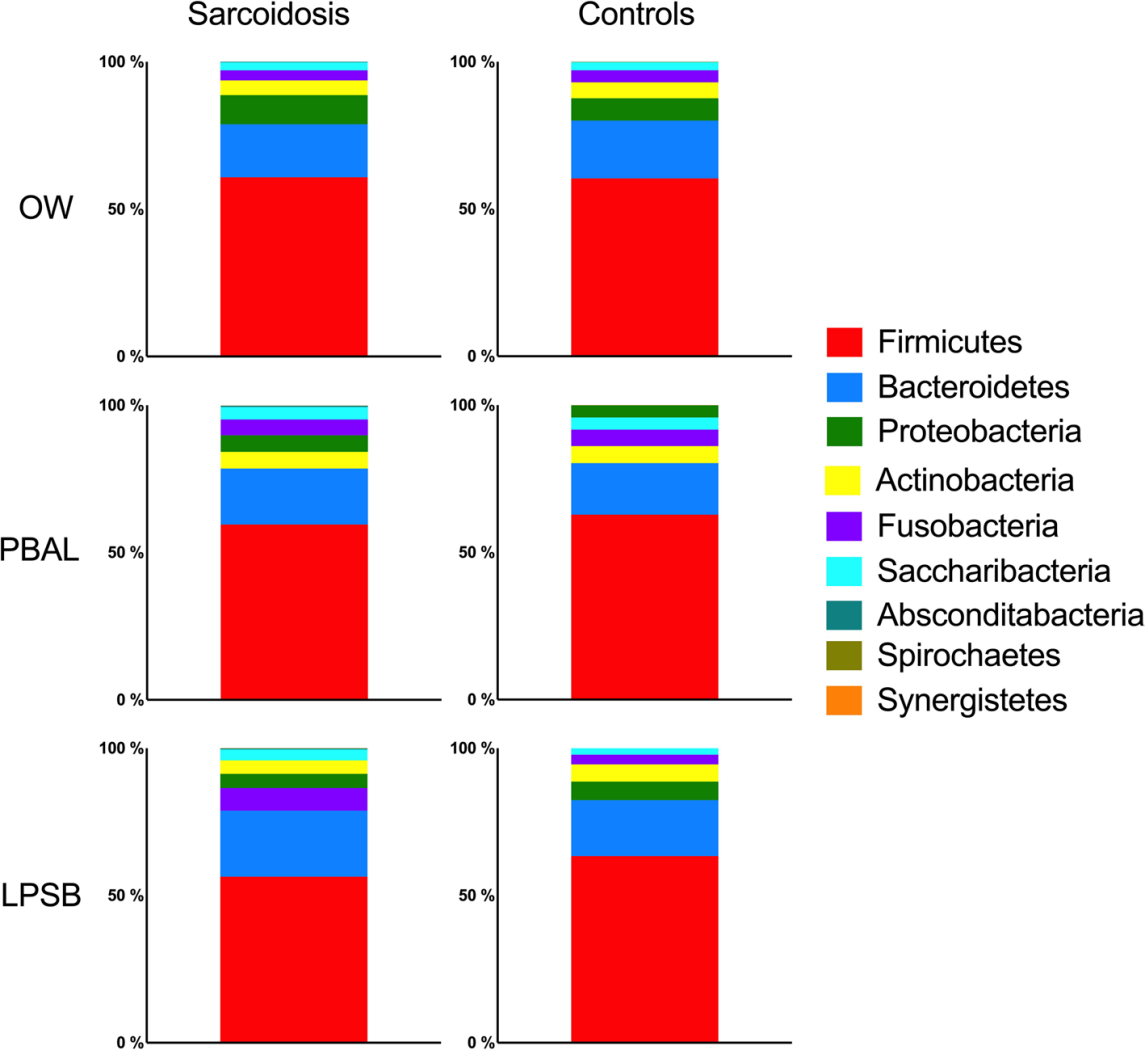
Bacterial taxonomy at phylum level by study groups and sample types (OW: oral wash, PBAL: protected bronchoalveolar lavage, LPSB: left protected sterile brushes)

**Fig. 4:**
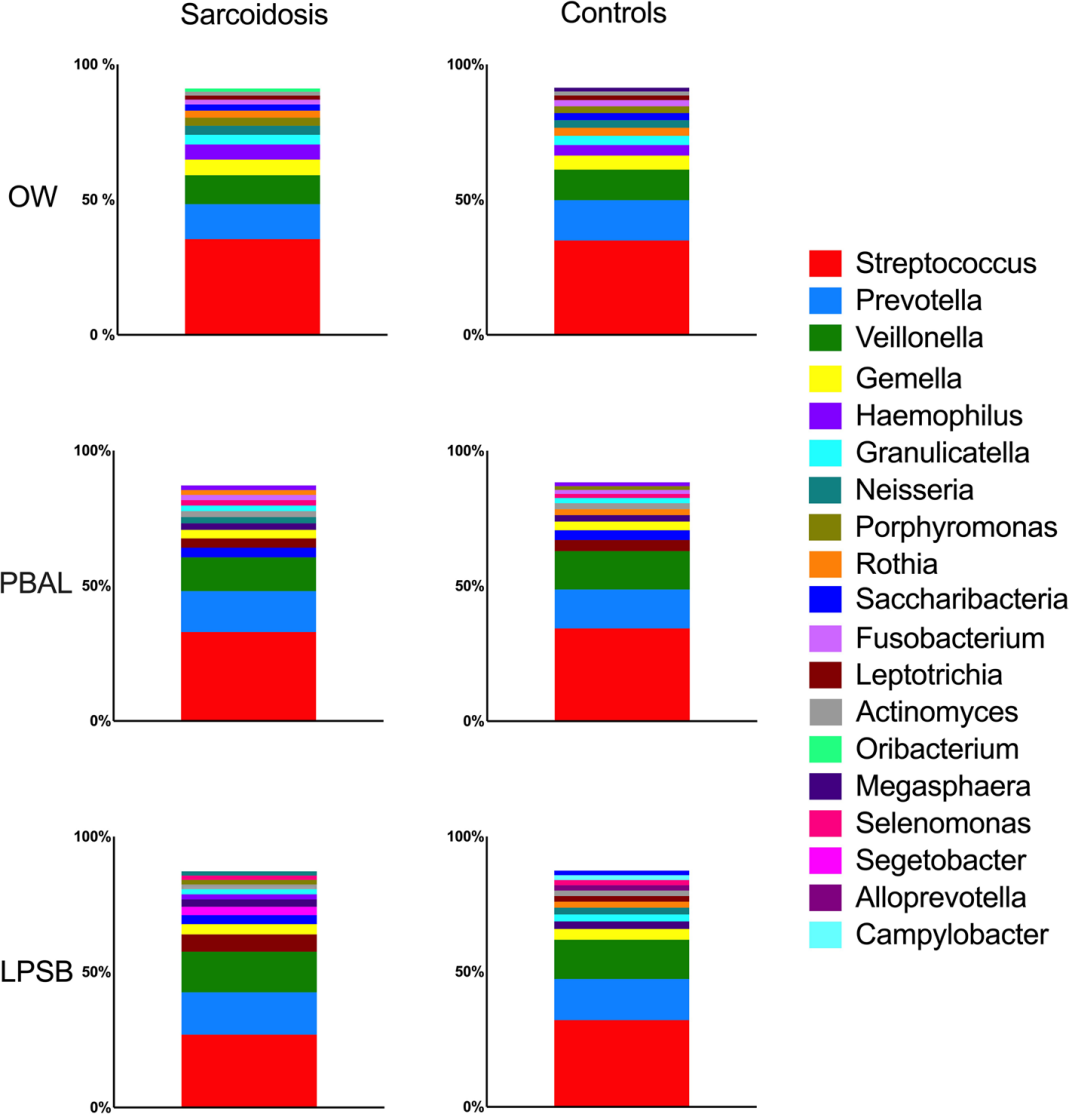
Bacterial taxonomy at genus level by study groups and sample types (OW: oral wash, PBAL: protected bronchoalveolar lavage, LPSB: left protected sterile brushes)

The distribution of the bacterial taxonomic composition for the different study groups and sample types at phylum and genus levels respectively are shown with stacked bar plots in Figs. 3 and 4. The taxa are visualized in the order of decreasing relative abundance for each category. *Firmicutes,* with *Bacteroidetes* coming second, dominated the phylum level. *Streptococcus, Prevotella*, and *Veillonella* dominated the genus level for both study groups. When assessing taxonomic differences (ANCOM), there were significantly less *Abscondibacteria* in PBAL and *Fusobacteria* in OW for sarcoidosis patients compared to controls at phylum level, and significantly more *Stomatobacteria* in OW and *Synergistia* in LPSB in patients compared to controls at genus level (Table 2).

### Diversity

For the fungal microbiota, no significant differences in alpha diversity were found between the study categories and sample types, measured with Shannon’s non-phylogenetic diversity (Table 3).

**Table 3:** Comparison of alpha diversity assessed by Faith’s phylogenetic diversity (PD) and Shannon’s non-phylogenetic diversity indexes for the different fungal and bacterial samples

Sarcoidosis vs controls	Fungi		Bacteria		
	OW^a^	PBAL^a^	OW^a^	PBAL^a^	LPSB^a^
Faith’s PD			0.047	0.03	0.35
Shannon	0.84	0.87	0.13	0.55	0.25

Numbers represent *p* values estimated from Kruskal-Wallis tests

^a^Oral wash (OW), protected bronchoalveolar lavage (PBAL), left protected sterile brushes (LPSB)

**Fig. 5:**
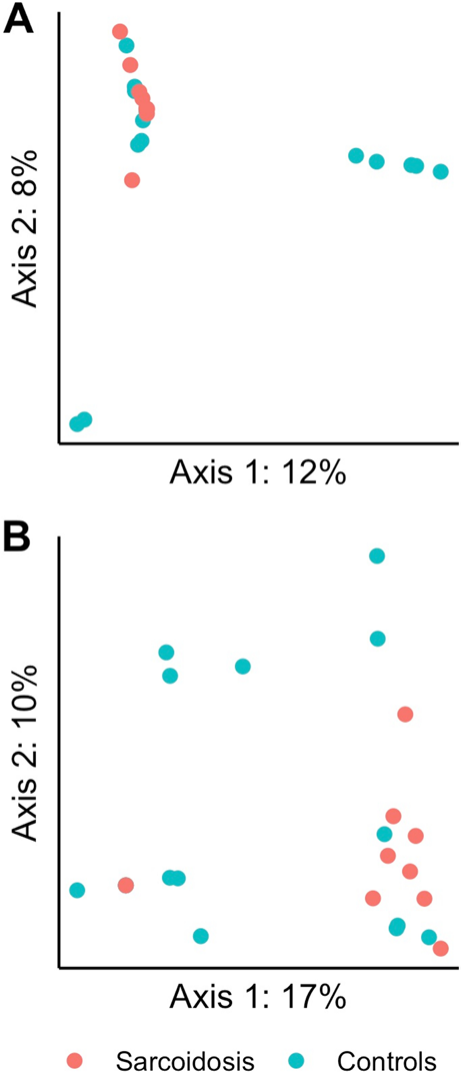
Principle coordinate analysis (PCoA) of fungal diversity in protected bronchoalveolar lavage (PBAL) by study groups. PERMANOVA (999 permutations) for the different distance matrices (**A** = Jaccard, **B** = Bray-Curtis) showed significantly more similar fungal beta diversity for sarcoidosis illustrated with red dots compared with controls illustrated with green dots

The beta diversity however differed significantly between sarcoidosis and controls as estimated in PBAL by Jaccard (*p* = 0.03) and Bray-Curtis (*p* = 0.03), and in OW by Jaccard (*p* = 0.04) (see Supplementary Table 1 in the Additional file 2). The principal coordinates analysis (PCoA) plots for the two beta diversities for PBAL samples are visualized in Fig. 5.

**Fig. 6:**
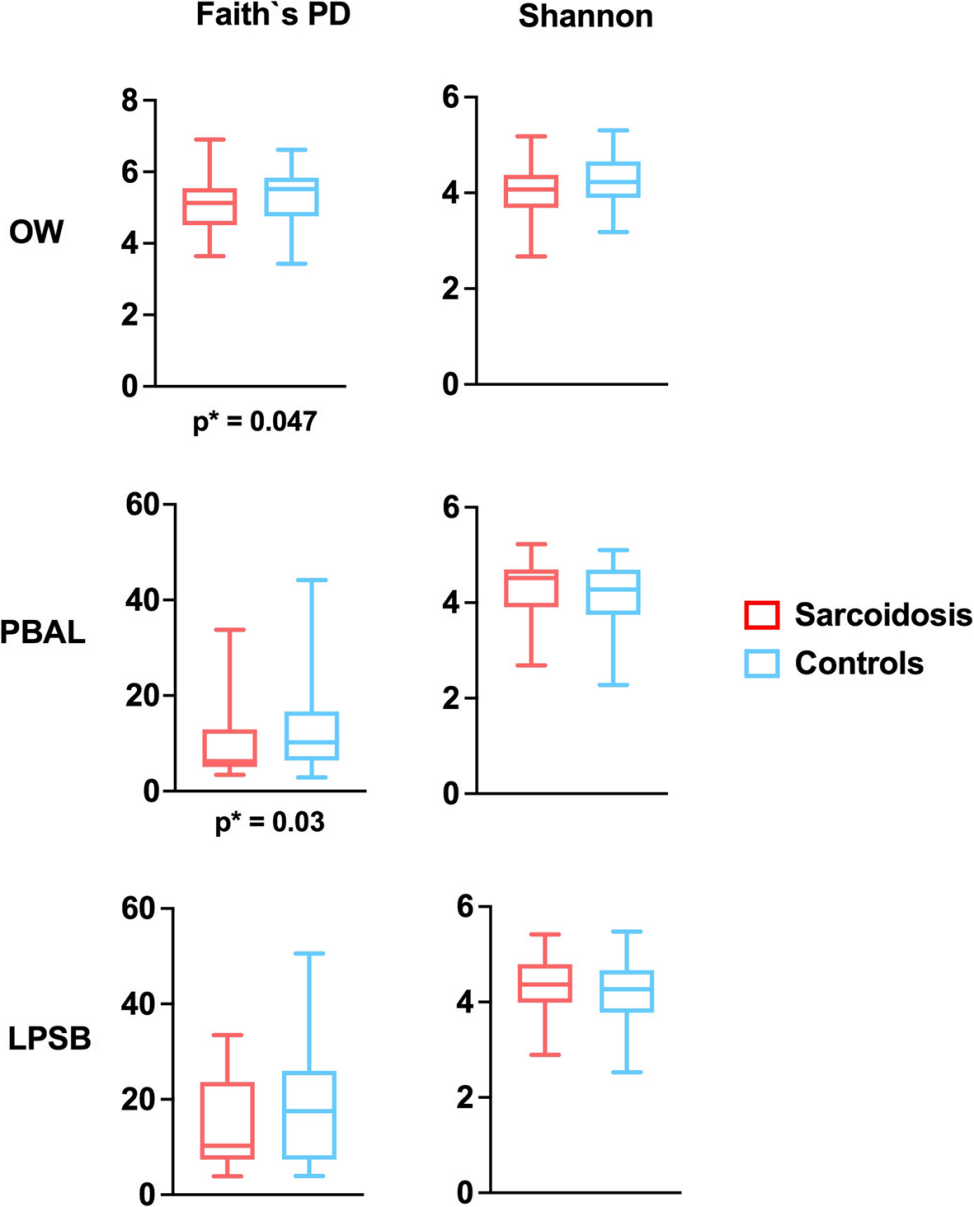
Bacterial alpha diversity measured with Faith’s phylogenetic diversity by study groups (sarcoidosis, controls) and sample types (OW: oral wash, PBAL: protected bronchoalveolar lavage, LPSB: left protected sterile brushes)

The bacterial microbiota in sarcoidosis patients was significantly less diverse, as measured by Faith’s phylogenetic alpha diversity in OW and PBAL, compared to controls (Fig. 6 and Table 3). There were no significant differences between the sample types for the study groups when tested with Shannon’s non-phylogenetic diversity (Table 3).

**Fig. 7:**
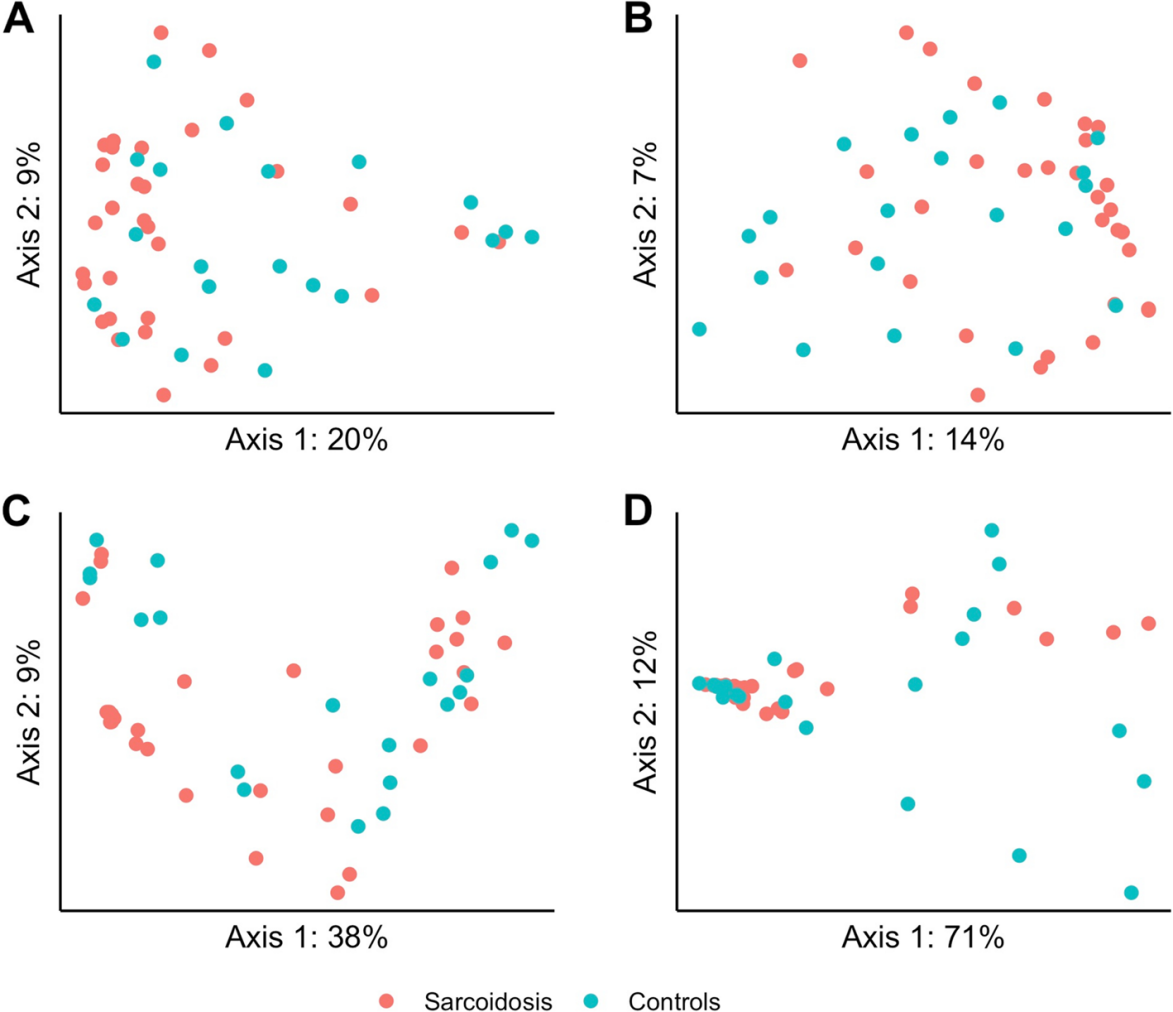
Principle coordinate analysis (PCoA) of bacterial beta diversity in left protected sterile brushes (LPSB) by study groups. PERMANOVA (999 permutations) for the different distance matrices (**A** = Jaccard, **B** = Bray-Curtis, **C** = weighted UniFrac, **D** = unweighted UniFrac) showed significantly more similar bacterial beta diversity for sarcoidosis illustrated with red dots compared with controls illustrated with green dots

The bacterial beta diversity differed significantly between sarcoidosis and controls as estimated in OW by Jaccard (*p* < 0.01), in PBAL by unweighted UniFrac (*p* = 0.02); and in LPSB by weighted UniFrac (*p* = 0.02), unweighted UniFrac (*p* = 0.03), Bray-Curtis (*p* < 0.01), and Jaccard (*p* < 0.01) (see Supplementary Table 1 in the Additional file 2). The PCoA plots for the different beta diversities for LPSB samples are shown in Fig. 7.

### Antimicrobial peptides and proteins

The levels of AMPs in the PBAL samples were significantly lower in sarcoidosis compared to controls for SLPI (*p* < 0.01) and hBD-1 (*p* < 0.01) (Table 1). However, we found no significant correlations between the bacterial or fungal alpha diversity and either of SLPI, hBD-1 or hBD-2 when testing with Spearman’s non-parametric correlation test (see Supplementary Table 2 in the Additional file 2).

## Discussion

This study demonstrated that stable sarcoidosis patients have a different fungal and bacterial microbial composition in the lower airways and oral cavity compared with healthy controls, being most pronounced for fungi. Differences between sarcoidosis and controls were found when examining differential abundances of taxonomy and differences in microbial diversity. The lower airways levels of the antimicrobial peptides SLPI and hBD-1 also differed between sarcoidosis and controls, with lower levels in sarcoidosis. However, we did not find a statistically significant association between the alpha diversity and the examined antimicrobial molecules.

Since the airways are not sterile in healthy individuals, a potential hypothesis is that a dysbiosis of the lower airways microbiota is a causal factor in the aberrant immune response seen in several chronic lung diseases. The role of the microbiome in the pathogenesis of pulmonal sarcoidosis has only been examined in a few studies, without consistent results. A recently published paper by Greaves et al. found enrichment of a peptide from *Aspergillus nidulans* when examining BAL fluid in 9 patients with Lofgren syndrome, compared with 3 controls, together with increased serum antibodies, and the authors suggested that *Aspergillus nidulans* had a potential pathogenic role [6]. Clarke and colleagues used a variety of sequencing strategies to look for key bacteria, fungi, and viruses in sarcoidosis when studying BAL fluid, lymph nodes and splenic tissue in a study of 93 sarcoidosis patients with different control subjects. They identified limited enrichment of *Aspergillus* (within the Eurotiales order) in BAL fluid but questioned this to be contamination [5]. In another study of the bacterial microbiota with 71 sarcoidosis patients, 15 IPF patients, and 10 healthy controls, BAL measurements revealed more *Fusobacterium* spp and *Atopobium* spp in sarcoidosis compared to healthy controls [8]. Fukui et al. compared lung microbiota between patients with sarcoidosis and anti-neutrophil cytoplasmic antibody-associated (ANCA) vasculitis, and found clustering of the *Erythrobacteraceae* family in sarcoidosis patients [11]. Gupta et al. performed a comparative analysis of the alveolar microbiome in 27 COPD patients, 8 ILD and 8 sarcoidosis patients and found *Actinobacteria* and *Proteobacteria* to be significantly more abundant in sarcoidosis patients compared to ILD patients [12]. Three other studies of the bacterial microbiota sampled with BAL in sarcoidosis have compared the bacterial composition in sarcoidosis patients with ILD patients. A study by Becker et al. with 31 sarcoidosis patients and 19 ILD patients [9], a study by D’Argenio et al. examining 10 sarcoidosis patients and 9 ILD patients [10], and a study by Garzoni et al. in a ILD cohort consisting of 11 interstitial pneumonia patients and 7 sarcoidosis patients compared with 6 *Pneumocystis* pneumonia patients and 9 healthy controls [7]. All three studies produced negative results, in that a distinct bacterial profile differentiating sarcoidosis from the other groups was not found. None of the above studies used protected BAL for sampling.

In our study, the differences between patients and controls were more striking for the fungal taxonomy compared with the bacterial taxonomy. *Candida* was the most abundant genus in the OW samples for both groups and in the PBAL samples from healthy controls*. Candida* was significantly lower in PBAL samples for sarcoidosis patients compared to controls, possibly driven by *Aspergillus*, which was the most frequent taxon in this group. This was in line with the two other known studies on the fungal pulmonary microbiota in sarcoidosis patients as previously described [5, 6]. *Candida* and *Aspergillus* are the most prevalent and well-known fungal pathogens [23]. Increased levels of antifungal antibodies in BAL fluid and serum have been found in sarcoidosis patients compared to controls, indicating that fungal infection can be a possible etiologic agent of sarcoidosis [24]. Antifungal therapy for sarcoidosis was tested in a small study [25], but needs much more work for confirmation. Some common fungal taxa identified in a healthy lung have been C*ladosporium* and *Penicillium* [23, 26], which also were present in our study.

The most abundant bacterial phyla we detected were *Firmicutes* and *Bacteroidetes*, while *Streptococcus*, *Prevotella*, and *Veillonella* dominated the genus level, all shared by both study groups. These taxa are described as the most common bacteria in both the lower and upper airways in a healthy lung [27]. Differential abundance testing with ANCOM revealed some statistically significant differences with infrequent microbes both at phylum and genus level, but it is uncertain whether this has clinical relevance.

Antimicrobial peptides are produced by immune and inflammatory cells in the lungs and other mucosal tissues, and exhibit broad spectrum antimicrobial, immune modulatory, and wound repair features [14]. These molecules are conserved as part of the regulation of the balance between a healthy microbiota and the innate immune system. AMPs are gaining increased attention as novel antimicrobial agents [28], but there are few scientific reports on how antimicrobial peptides interact and respond to the aberrant immunologically activity in sarcoidosis. AMPs display a wide range of antifungal activities [29], also shown for SLPI [30]. In addition to antimicrobial activity, SLPI is an important antiprotease in the lungs. Sarcoidosis patients with reduced lung function have higher levels of TGF-β in BAL [31], which again are linked to lower levels of SLPI [32].

Interestingly, we found significantly lower values for SLPI and hBD-1 in our study for sarcoidosis patients compared to controls. Agerberth et al. [15] examined antibacterial components in BAL fluid from 12 sarcoidosis patients and 10 healthy controls and found enhanced levels in sarcoidosis patients (among them SLPI, hBD-1 and 2). This was opposite from our results, but sub-analyses in Agerberth’s study found lower values of antibacterial components in sarcoidosis patients with inactive disease, than in those newly being treated with steroids. Our sarcoidosis patients were in a stable state and thus more likely to have inactive disease, so these observations are in line with our results.

Since our study is cross-sectional, we cannot differentiate between lower levels of anti-microbial peptides leading to a microbial dysbiosis, or vice versa. Low levels of antimicrobial peptides in the airways could be a pathogenic factor, or a sign of an overwhelmed innate immune response. In any event, the persistence of granuloma and inflammation in sarcoidosis point to activation of stimulating antigens driving the disease process, and our study indicates that the fungal microbiota may be part of this process.

Accurately measuring the low biomass microbiota in the lower airways is challenging. The bacterial DNA density in the upper airways is at least 100-fold higher than in the lower airways [33]. Low biomass samples are vulnerable to contamination during sampling and laboratory processing [34]. We therefore standardized protected sampling of the lower airways to minimize contamination from the upper airways [35], included sterile BAL catheters not usually employed by other studies, and used the validated Decontam method in the MicroCOPD study to identify contaminants [36]. Further, all our study samples are from one center, with consistent methodology from bronchoscopy to wet-lab and bioinformatic pipeline. Finally, we have included sampling from two different locations in the lungs, and from healthy controls, which most studies lack.

However, our study has some shortcomings. First, BAL yield will differ within both the patients and control groups, which could influence microbial composition and measured levels of the AMPs. Mean BAL yield was 47.2% (SD 11.6) for controls and 48.3% (SD 17.7) for the patients, thus reasonably similar but with larger spread for the patients. Second, our study lacks patients with acute sarcoidosis in the early stage of disease. Third, fungi are even more delicate to examine with next-generation sequencing compared to bacteria due to its low abundance in many samples, high levels of fungal DNA from contamination sources and finally lack of standardization in primer designs, reference databases, and analytic methods [26, 37]. The results we find are only as good as the method and databases per now. In the future, whole genome sequencing (WGS) should provide better species characterization. Fourth, this study lacks qPCR and thereby quantification of bacterial load. Absolute bacterial or fungal abundance could very well be important in disease development, but this study cannot address whether that is the case. Also, differences between study groups for absolute abundance cannot be tested, and therefore significant differences may have been missed. Fifth, this is a cross-sectional study and there is a need for longitudinal studies that look at microbiota changes over time and its implications for disease development. Finally, our analysis of antimicrobial peptides and proteins was limited to SLPI, hBD-1, and hBD-2. Whereas SLPI is present in high concentrations, including measurement of other abundant antimicrobials such as lysozyme, or antimicrobial peptides present in lower amounts (such as LL-37), could have contributed further insight, but was outside the scope of the present study. In addition, future studies may also include measurement of other antimicrobial components, such as antifungal chitinases.

## Conclusions

In this study, we found differences in fungal and bacterial diversity between sarcoidosis patients and controls, and a more clearly distinct fungal taxonomy in the lower airways compared with controls, where *Aspergillus* genera dominated the lower airways in sarcoidosis. In addition, sarcoidosis patients had lower levels of antimicrobial peptides in the airways. These findings could indicate the presence of a microbial dysbiosis in the airways in sarcoidosis. Future research should address whether this dysbiosis has a pathogenic role, and thus be a potential target for new treatment principles.

## Supplementary Information

**Additional file 1.** Supplementary Methods.

**Additional file 2: Supplementary Table 1**. Comparison of beta diversity by pairwise PERMANOVA between the sarcoidosis patients and controls for fungal and bacterial taxa sampled by different methods* and assessed by different beta diversity metrices. **Supplementary Table 2**. Spearman's correlation coefficients (rho) and p-values for differences in alpha diversity correlated with antimicrobial peptides.

## Data Availability

All required data files have been uploaded to DRYAD and will be shared as part of our commitment to open science. 10.5061/dryad.sn02v6x60
