# Supplementary material for: The lower airways microbiota and antimicrobial peptides indicate dysbiosis in sarcoidosis
Source: Microbiome. 2022 Oct 19;10:175. doi: 10.1186/s40168-022-01362-4 (PMC9580159; doi:10.1186/s40168-022-01362-4)
Supplement: Supplementary file 2 — Additional file 1. Supplementary Methods. [file 40168_2022_1362_MOESM1_ESM.docx]

**SUPPLEMENATARY METHODS**

***DNA extraction and PCR***

DNA was extracted with an enzymatic lysis with lysozyme, mutanolysin and lysostaphin, and mechanical lysis using the FastPrep-24 as described by the manufacturers of the FastDNA Spin Kit (MP Biomedicals, LLC, Solon, OH, USA).

For the fungal microbiota, the internal transcribed spacer (ITS) 1 region was PCR amplified using primer set ITS1-30F/ITS1-217R, with sequences GTCCCTGCCCTTTGTACACA and TTTCGCTGCGTTCTTCATCG[1]. Samples were paired-end sequenced on Illumina HiSeq (Illumina Inc., San Diego, CA, USA). LPSB was not sequenced for fungi.

For the bacterial microbiota, the V3V4 region of the 16S rRNA gene was PCR amplified. The DNA was quantified, normalized and sequenced using the protocol for 16S Metagenomic Sequencing Library Preparation for the Illumina MiSeq System. To adjust for errors, a mock-community and control samples of PBS fluid were included[2].

***Bioinfomatics analyses***

QIIME 2 was used for bioinfomatics analyses [3].

For the fungal microbiota:

Amplicon sequences were quality controlled (via q2-demux) and trimmed using the q2-itsxpress plugin. Divisive Amplicon Denoising Algorithm 2 (DADA2) removed low quality reads, chimeras and created the exact amplicon sequence variants (ASVs), which were curated using the Lulu R package[4] to remove artefactual ASVs. The Decontam package in R removed contaminants, and taxonomy was assigned with a UNITE (https://unite.ut.ee) database for fungi[5].

For the bacterial microbiota:

Amplicon sequences were demultiplexed using q2-demux plugin and then denoised with DADA2. Chimeras were removed with DADA2 and VSEARCH. All ASVs were processed with removal of contaminants with the prevalence based approach in the Decontam[6] package in R assigned with taxonomy using the Human Oral Microbiome Database (HOMD)[7] and aligned with mafft[8].

Diversities for both the fungal and bacterial microbiota were estimated using the q2-diversity plugin after samples were rarefied to 1000 sequences per sample. We choose a rarefaction depth as high as possible while excluding a minimum of samples. The fungal microbiota diversity analyses did not incorporate the phylogenetic relationships between the species, as alignments of ITS sequences are not useful for informing evolutionary distances among distantly related species.

For the statistical analyses including ANCOM and PERMANOVA we used QIIME 2[3].

**REFERENCES**

1. Usyk M, Zolnik CP, Patel H, Levi MH, Burk RD. Novel ITS1 Fungal Primers for Characterization of the Mycobiome. mSphere. 2017;2

2. Drengenes C, Eagan TML, Haaland I, Wiker HG, Nielsen R. Exploring protocol bias in airway microbiome studies: one versus two PCR steps and 16S rRNA gene region V3 V4 versus V4. BMC Genomics. 2021;22:3.

3. Bolyen E et al. Reproducible, interactive, scalable and extensible microbiome data science using QIIME 2. Nat Biotechnol. 2019;37:852-857.

4. Frøslev TG et al. Algorithm for post-clustering curation of DNA amplicon data yields reliable biodiversity estimates. Nat Commun. 2017;8:1188.

5. (2019) UNITEC. UNITE QIIME release for Fungi. Version 18.11.2018. UNITE community. https://doi.org/10.15156/BIO/786334.

6. Davis NM, Proctor DM, Holmes SP, Relman DA, Callahan BJ. Simple statistical identification and removal of contaminant sequences in marker-gene and metagenomics data. Microbiome. 2018;6:226.

7. Chen T, Yu WH, Izard J, Baranova OV, Lakshmanan A, Dewhirst FE. The Human Oral Microbiome Database: a web accessible resource for investigating oral microbe taxonomic and genomic information. Database (Oxford). 2010;2010:baq013.

8. Katoh K, Misawa K, Kuma K, Miyata T. MAFFT: a novel method for rapid multiple sequence alignment based on fast Fourier transform. Nucleic Acids Res. 2002;30:3059-3066.
