# Supplementary material for: The lower airways microbiota and antimicrobial peptides indicate dysbiosis in sarcoidosis
Source: Microbiome. 2022 Oct 19;10:175. doi: 10.1186/s40168-022-01362-4 (PMC9580159; doi:10.1186/s40168-022-01362-4)
Supplement: Supplementary file 3 — Additional file 2: Supplementary Table 1. Comparison of beta diversity by pairwise PERMANOVA between the sarcoidosis patients and controls for fungal and bacterial taxa sampled by different methods* and assessed by different beta diversity metrices. Supplementary Table 2. Spearman's correlation coefficients (rho) and p-values for differences in alpha diversity correlated with antimicrobial peptides. [file 40168_2022_1362_MOESM2_ESM.docx]

| **SUPPLEMENTARY DATA**  **Supplementary Table 1. Comparison of beta diversity by pairwise PERMANOVA between the sarcoidosis patients and controls for fungal and bacterial taxa sampled by different methods* and assessed by different beta diversity metrices** | | | | | |
| --- | --- | --- | --- | --- | --- |
| **Sarcoidosis vs Controls** | **Fungi** | | **Bacteria** | | |
|  | OW* | PBAL* | OW* | PBAL* | LPSB* |
|  |  |  |  |  |  |
| Bray-Curtis | 0.41 | 0.03 | 0.47 | 0.43 | <0.01 |
| Jaccard | 0.04 | 0.03 | <0.01 | 0.06 | <0.01 |
| Weighted UniFrac |  |  | 0.41 | 0.44 | 0.02 |
| Unweighted UniFrac |  |  | 0.17 | 0.02 | 0.03 |
| * Oral wash (OW), protected bronchoalveolar lavage (PBAL), left protected sterile brushes (LPSB) | | | | | |
|  |  |  |  |  |  |

| **Supplementary Table 2. Spearman's correlation coefficients (rho) and p-values for differences in alpha diversity correlated with antimicrobial peptides** | | | | | | | | | | | | | |
| --- | --- | --- | --- | --- | --- | --- | --- | --- | --- | --- | --- | --- | --- |
|  |  | **hBD-1†** | | | | **hBD-2†** | | | | **SLPI†** | | | |
|  |  | Faith`s PD | | Shannon | | Faith`s PD | | Shannon | | Faith`s PD | | Shannon | |
|  | **Sample type** | *rho* | *p* | *rho* | *p* | *rho* | *p* | *rho* | *p* | *rho* | *p* | *rho* |  |
| **Fungi** | OW* |  |  | 0.13 | 0.36 |  |  | -0.02 | 0.90 |  |  | 0.006 |  |
|  | PBAL* |  |  | 0.07 | 0.74 |  |  | 0.20 | 0.31 |  |  | 0.19 |  |
| **Bacteria** | OW* | 0.14 | 0.18 | 0.11 | 0.30 | 0.07 | 0.48 | 0.09 | 0.40 | 0.14 | 0.20 | 0.14 |  |
|  | PBAL* | 0.05 | 0.68 | 0.19 | 0.08 | -0.05 | 0.64 | 0.08 | 0.48 | -0.001 | 1.0 | 0.14 |  |
|  | LPSB* | 0.02 | 0.88 | 0.13 | 0.29 | 0.22 | 0.07 | 0.19 | 0.12 | 0.01 | 0.93 | 0.14 |  |
| * Oral wash (OW), protected bronchoalveolar lavage (PBAL), left protected sterile brushes (LPSB) | | | | | | | | | | |  |  |  |
| † human beta defensins 1 and 2 (hBD-1 & hBD-2), Secretory leucocyte protease inhibitor (SLPI) | | | | | | | | | | |  |  |  |
